# Mitigating Pro‐Inflammatory SASP and DAMP With Urolithin A: A Novel Senomorphic Strategy

**DOI:** 10.1111/acel.70237

**Published:** 2025-09-29

**Authors:** Anna Barkovskaya, Ashley Brauning, Aditi Thambala, Kriti Bhardwaj, Manish Chamoli, Anand Rane, Julie K. Andersen, Amit Sharma

**Affiliations:** ^1^ Lifespan Research Institute Mountain View California USA; ^2^ Buck Institute for Research on Aging Novato California USA

**Keywords:** cGAS‐STING signaling, cytosolic DNA, senescence, senomorphic, Urolithin A

## Abstract

Senescent cells are known to contribute to aging and age‐related diseases. One key way they influence aging is by secreting senescence‐associated secretory phenotype (SASP) factors along with several damage‐associated molecular pattern (DAMP) molecules. Consequently, inhibiting SASP and DAMP signaling (senomorphics) has emerged as a therapeutic strategy. Urolithin A (UA), a gut‐derived metabolite produced from ellagitannins and ellagic acid found in berries, nuts, and pomegranates, has demonstrated potent anti‐inflammatory properties and protective effects against aging and age‐related conditions in experimental models. Here we demonstrate that UA lowers the expression and release of pro‐inflammatory SASP and DAMP factors, at least in part, by downregulating cytosolic DNA release and subsequent decrease in cGAS‐STING signaling.

## Graphical abstract

Senescent cells accumulate extranuclear DNA fragments that are recognized by the cyclic GMP‐AMP synthase (cGAS) and its effector protein stimulator of interferon genes (STING), which promotes expression of SASP and DAMP genes by inducing nuclear factor‐kappa B (NF‐κB) and IFN regulatory factor 3 (IRF3) translocation. UA inhibits this process by preventing cytosolic DNA leakage and reducing cGAS‐STING signaling.

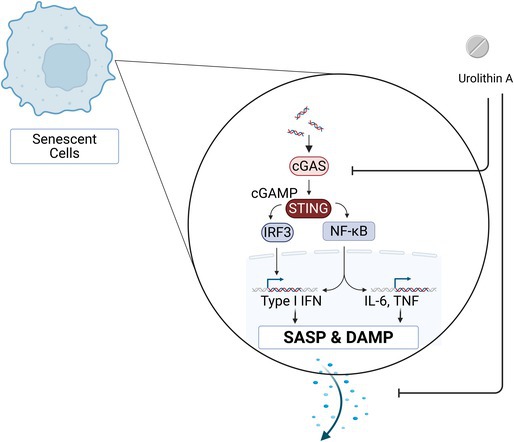

## 1 Introduction

Aging is associated with increased systemic sterile inflammation (inflammaging), which promotes several age‐associated diseases (Franceschi and Campisi 2014). Key drivers of inflammaging include senescence‐associated secretory phenotype (SASP) factors released by senescent cells (Olivieri et al. 2018). While the exact components of SASP vary between different senescent cells and tissues, core SASP factors include pro‐inflammatory chemokines, matrix‐degrading enzymes, and several damage‐associated molecular pattern (DAMP) molecules (Coppe et al. 2010). Pharmacological inhibition of SASP using small molecules known as senomorphics has been proposed as a potential intervention for age‐associated diseases (Kim and Kim 2019; Lagoumtzi and Chondrogianni 2021). However, such treatments include several flavonoid inhibitors of the p38 MAPK/NF‐κB pathway (Freund et al. 2011; Zhang et al. 2023), free radical scavengers, and Janus kinase (JAK) pathway inhibitors (Niedernhofer and Robbins 2018) that are nonselective and broadly inhibit pathways also involved in homeostatic immune responses to various physiological challenges, thus limiting their systemic therapeutic application (Zhang et al. 2017).

Here we present data indicating that the gut metabolite Urolithin A (UA) acts as a senomorphic compound. Digestive tract bacteria naturally produce UA through the metabolism of ellagitannins and ellagic acid, which are abundant in berries, nuts, and pomegranates (D'Amico et al. 2021). UA has been reported to be a potent anti‐inflammatory agent, alleviating several age‐related conditions in vivo (D'Amico et al. 2021; Ishimoto et al. 2011; Larrosa et al. 2010). Preclinical studies have also shown its protective role against aging and age‐related conditions affecting the muscles, brain, joints, and other organs (D'Amico et al. 2021). In a recent clinical trial, UA supplementation improved muscular endurance in older adults (Liu et al. 2022).

## 2 Results and Discussion

**FIGURE 1:**
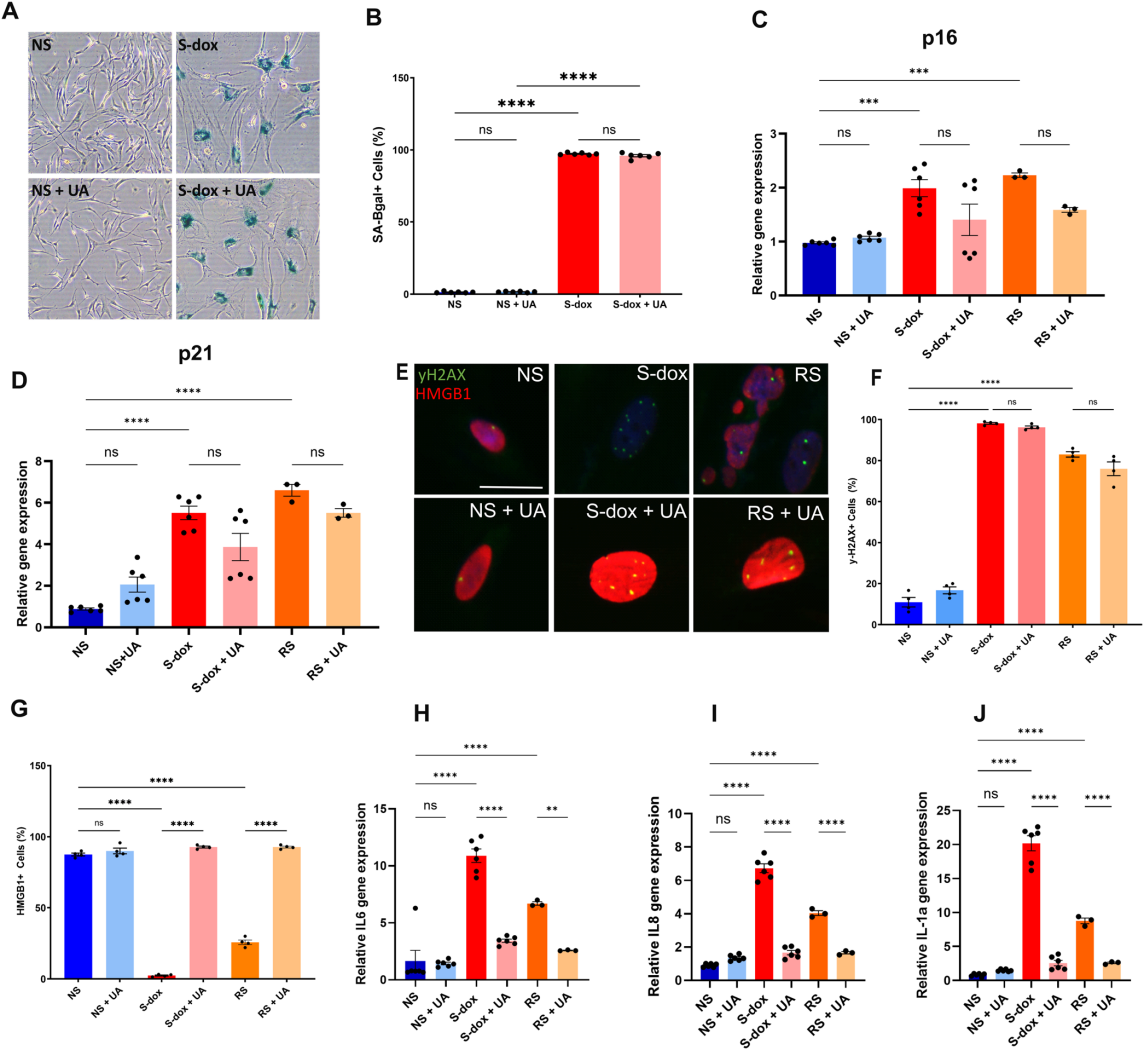
Doxorubicin treatment induces senescence in human IMR‐90 fibroblasts. (A) Representative images of SA‐βGal‐stained S‐dox and NS with or without the addition of Urolithin A (UA). (B) The proportion of cells staining positive for SA‐βGal in NS and S‐dox cells with and without the UA treatment. Four fields were quantified per well (*n* = 3). (C, D) Relative p16 (C) and p21 (D) expression in NS, S‐dox, and RS cells with and without UA treatment, *n* = 4. (E) IF staining for γ‐H2AX and HMGB1 in NS, S‐dox, and RS cells with or without the addition of UA, 10 days post‐induction. γ‐H2AX—green, HMGB1—red, and DAPI—blue. Scale bar = 30 μm. (F, G) The percentage of cells with two or more γ‐H2AX foci per cell (F) and with nuclear HMGB1 (G). (H–J) Fold change of IL6 (H), IL8 (I), and IL1α (J) gene expression in S‐dox and RS cells relative to NS control. *n* = 4. All data are presented as mean ± SEM. One‐way ANOVA. **p < 0.002, ***p < 0.0002 ****p < 0.0001.

To test the effect of UA on senescent cells, we induced senescence in the human fetal lung fibroblast IMR‐90 line via treatment with 300 nM doxorubicin (S‐dox). We observed robust senescence induction as measured by senescence‐associated beta‐galactosidase (SA‐β‐gal) staining (93%) 10 days following doxorubicin treatment compared to non‐senescent controls (< 2%) (Figure 1A,B). We confirmed this in two additional fibroblast cell lines (Figure S1A,B). In addition to chemotherapy‐induced senescence, we also examined the effects of UA in the context of replicative senescence (RS) (Campisi 1997). Both doxorubicin‐treated S‐dox and RS cells displayed significantly elevated expression of the cell cycle checkpoint inhibitors *p16* ^*INK4A*^ and *p21* ^*CIP*^. Treatment with UA had no significant effect on the expression of these markers (Figure 1C,D). Previously, UA pre‐treatment has been shown to reduce the percentage of SA‐β‐gal expressing cells and p53 and p21 expression in senescent Organ of Corti 1 (HEI‐OC1) cells and cochlear explants (Cho et al. 2022) or mesenchymal stem cells derived from nucleus pulposus (Cho et al. 2022; Shi et al. 2021). In another study, treatment with UA did not affect the expression of SA‐β‐gal in human skin fibroblasts undergoing replicative senescence (Liu et al. 2019). While our results show no significant effect on the expression of *p16* ^*INK4A*^ and *p21* ^*CIP*^, albeit with a trend towards lower expression, it may be due to the diversity of senescence phenotypes. However, UA did not reduce cell viability in either the proliferating IMR‐90 or the S‐dox in the tested concentration range (Figure S1C). It is important to note that while UA did not have any senolytic effect, senescence is heterogeneous and in other cell types, or modes of senescence induction, UA may induce cytotoxicity. In particular, UA has been shown to stimulate autophagy (Leng et al. 2013; Xavier et al. 2013). In senescent cells with extensive mitochondrial dysfunction and dysregulation, this can lead to autophagy‐induced cell death.

Further confirming senescence induction, most doxorubicin‐treated IMR‐90 cells had two or more γH2AX foci, indicating activation of the DNA‐damage response (DDR) triggered by the double‐strand DNA breaks (Hernandez‐Segura et al. 2018; Kinner et al. 2008). The number of individual foci has been used as an indication of the severity of the DNA damage (Noubissi et al. 2021). Even though treatment with UA did not reduce the proportion of cells with the activated DDR (Figure 1E,F), we observed fewer foci in UA‐treated senescent cells. However, it did not reach statistical significance in either the NS or the S‐dox cells (Figure S1D). The loss in the nuclear localization of high mobility group box 1 (HMGB1), a member of the highly conserved nonhistone DNA‐binding high‐mobility group protein family, is a well‐established senescence marker (Davalos et al. 2013). Its secretion from senescent cells has been reported as part of DAMP signaling, a key driver of the senescent phenotype (Wiley and Campisi 2021). Notably, treatment with UA significantly reduced the percentage of cells that lost HMGB1 from the nucleus in both the doxorubicin‐treated and RS cells (Figure 1E,G).

**FIGURE 2:**
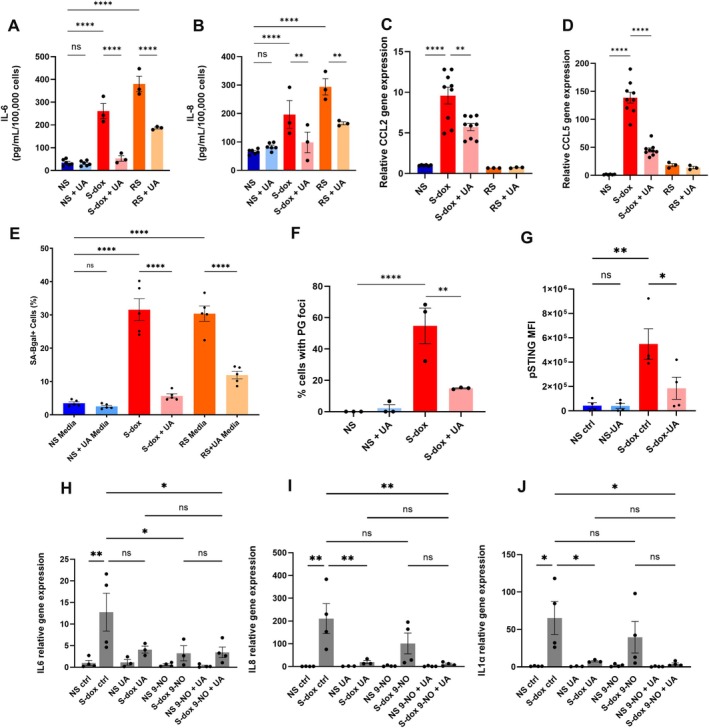
UA decreases paracrine senescence through downregulation of c–GAS–STING signaling. (A, B) ELISA on conditioned media collected from NS, S‐dox, and RS cells with and without UA treatment after 24 h of incubation. IL‐6 (A) and IL‐8 (B) cytokine concentrations were normalized to 100,000 cells, *n* = 3. (C, D) Relative gene expression of CCL2 (C) and CCL5 (D) in NS, S‐dox, and RS cells with or without UA treatment, *n* = 3. (E) Proportion of SA‐βGal‐positive cells 6 days after they have been treated with media collected from NS, S‐dox, or RS cultured with or without UA for 6 days, *n* = 3. (F) Proportion of cells containing extranuclear pico green (PG)—stained DNA foci in NS and S‐dox cells with or without UA treatment. *n* = 3. (G) Quantification of the pSTING staining intensity in the NS and S‐dox cells, with and without UA treatment, *n* = 3. (H–J) Fold change in IL6 (H), IL8 (I), IL1α (J) gene expression in NS and S‐dox cells treated with UA, 9‐NO, or a combination of both. *n* ≥ 3. All data are presented as mean ± SEM. One‐way ANOVA. *p < 0.033, **p < 0.002, ****p < 0.0001.

Treatment with UA also resulted in a significant reduction in the expression of prototypical SASP factors, including interleukin 6 (*il6*), interleukin 8 (*il8*), and interleukin 1‐alpha (*il1α*), in both models of senescence induction, with no effect on the NS cells (Figure 1H–J, Figure S1E,F). Reduced gene expression was followed by a significant decrease in the secretion of IL‐6 and IL‐8 after UA treatment, as measured by ELISA in both doxorubicin‐treated and replicative senescent cells (Figure 2A,B).

The primary physiological function of SASP from senescent cells is to recruit immune cells, facilitating tissue repair, and regeneration during wound healing. The pro‐inflammatory chemokines C‐C motif ligand 2 (CCL2) and C‐C motif chemokine ligand 5 (CCL5) can recruit and activate macrophages and NK cells, promoting local inflammation (Budamagunta et al. 2021; Luciano‐Mateo et al. 2020). As expected, expression of both chemokines was significantly upregulated in senescent cells. Treatment with UA mildly reduced expression, specifically in doxorubicin‐treated cells, suggesting that UA has a distinct mechanism of action dependent on the type of senescence induction (Figure 2C,D).

We have previously demonstrated that, in addition to their pro‐inflammatory function, SASP factors induce senescence in neighboring cells (Admasu et al. 2023). As UA reduced the levels of IL‐6 and IL‐8 in the conditioned media (CM), we tested if its treatment abrogates paracrine senescence by culturing proliferating IMR‐90 in the presence of media collected from either control or UA‐treated senescent cells. Our results showed significantly fewer SA‐β‐gal‐positive cells when treated with CM from UA‐treated senescent cells compared to CM from vehicle‐treated senescent cells, suggesting that it acts as an inhibitor of paracrine senescence (Figure 2E). While UA might have a broader impact on other SASP factors, its effect on the expression of IL‐6 and IL‐8 at least partially explains the ability of UA to reduce paracrine senescence, as both these cytokines are causally linked to paracrine senescence (Acosta et al. 2013; Kojima et al. 2013; Ortiz‐Montero et al. 2017; Li et al. 2020).

Finally, we examined the possible mechanisms associated with UA‐mediated regulation of cytokines and chemokines. Cellular senescence is associated with the accumulation of cytosolic DNA released from structurally impaired nuclei and mitochondria. Through an evolutionarily conserved mechanism of pathogen detection, misplaced DNA fragments are recognized by intracellular DNA sensors, including cyclic GMP‐AMP Synthase (cGAS) and the cGAS effector protein Stimulator of Interferon Genes (STING) (Sun et al. 2013). cGAS binding to double‐stranded DNA (dsDNA) results in cyclic guanosine monophosphate–adenosine monophosphate (cGMP) buildup as a secondary messenger that binds to and activates STING. STING, in turn, induces the translocation of transcription factors nuclear factor‐kappa B (NF‐κB) and IFN regulatory factor 3 (IRF3) into the nucleus (Sun et al. 2013). This promotes the expression of pro‐inflammatory SASP factors, which is further fueled by the direct exchange of cGAMP between neighboring cells through cGAMP transporters and gap junctions, thereby creating a more widespread inflammation (Loo et al. 2020; Aguado et al. 2021; Chen et al. 2016). The cGAS‐STING pathway has been proposed as a potential target for pharmacological intervention in disease treatments and controlling inflammation and immunity (Chin et al. 2023). We found that doxorubicin‐treated senescent cells indeed showed evidence of cytosolic DNA accumulation as demonstrated by induction of γH2AX foci (Figure S1G) and dsDNA‐specific pico green (PG) staining present outside of the nuclear boundaries (Figure 2F, Figure S1H). STING phosphorylation at the Ser366 residue was previously shown to be required for STING‐dependent activation of IRF3 downstream (Liu et al. 2015). Our results show that pSTING is significantly more abundant in senescent cells, reaching its highest levels shortly after treatment with doxorubicin, before returning to baseline by Day 7 (Figure S1I,J), while UA treatment downregulates pSTING in senescent cells (Figure 2G). Furthermore, we found that the cytosolic DNA foci were significantly reduced following treatment with UA, a finding consistent across all three cells we tested (Figure 2F, Figure S1K,L). These data suggest that UA may reduce the leakage of dsDNA, which aligns with previous reports demonstrating that UA enhances cytosolic DNA clearance in cancer cell lines (Madsen et al. 2024). Finally, to confirm that UA acts by regulating the cGAS‐STING signaling pathway, we tested a combination of UA treatment with a STING inhibitor 9‐nitro‐oleic acid (9‐NO), which inhibits STING palmitoylation at cysteines 88 and 91 (C88/91) that are crucial for STING activation (Hansen et al. 2018). The 9‐NO treatment downregulated expression of the SASP factors (*il6, il8, and il1α*). Co‐treatment with UA did not have any additional effect on this inhibition, suggesting that UA and STING regulate target gene expression through the same pathway (Figure 2H–J).

In aged mice, UA supplementation improved muscle strength, reduced inflammation, and promoted autophagic and mitochondrial health (Luan et al. 2021). UA treatment has also been shown to increase self‐renewal and prolong the survival of T memory stem cells (TSCM), thereby preventing T cell exhaustion and promoting effective anti‐tumor T cell responses, further supporting its use as an anti‐aging therapeutic (Luan et al. 2021). Senescent cells are known to have dysfunctional mitochondria, which results in oxidative stress. Over time, damaged mitochondria can release mitochondrial DNA (mtDNA) into the cytosol. Cytosolic mtDNA can act as a DAMP molecule, triggering inflammation through pathways such as the cGAS‐STING and the secretion of the SASP and DAMP. UA is known to activate mitophagy, so UA treatment may reduce cytosolic DNA by activating mitophagy in senescent cells.

Additionally, while cGAS‐STING activation primarily serves as an inflammatory response, it can also act as a feedback mechanism to promote mitochondrial quality control by activating mitophagy. Studies have demonstrated that cGAS‐STING activation can upregulate mitophagy‐related genes or enhance mitophagy activity through phosphorylation of autophagy adaptors by TBK1. Upregulation of mitophagy may reduce the accumulation of cytosolic DNA, inflammation, oxidative stress, and mitochondrial dysfunction (Jiménez‐Loygorri et al. 2024). UA has been previously shown to regulate mitophagy, accelerating mitochondrial turnover (Ryu et al. 2016; Toney et al. 2019; D'Amico et al. 2021). To test whether UA restores the mitochondrial integrity and function in senescent cells, we measured the proportion of mitochondria with a higher membrane potential, indicative of a functional electron‐transport chain and ATP production. However, UA did not significantly alter the proportion of active mitochondria in senescent fibroblasts, indicating a different mechanism at play (Figure S1M). It is likely that UA‐mediated regulation of mitophagy is context‐dependent as some of the insults triggering senescence, such as ultraviolet (UVA) radiation, are known to have a more severe effect, disrupting autophagy more generally (Xie et al. 2022; Ahsan et al. 2019). Taken together, these results suggest that UA may mitigate senescence‐associated damage and the subsequent release of DNA into the cytosol by distinct routes in context‐dependent ways, which may rely in part on the preservation of structural integrity of the nuclei and the mitochondria, as well as on the stimulation of autophagy.

In conclusion, these data demonstrate that UA's senomorphic effect is mediated by reducing cytosolic DNA and cGAS‐STING‐mediated inflammatory response, thus targeting the root cause of the condition, DAMP signaling.

## 3 Methods

### 3.1 Cell Culture and Urolithin A Treatment

IMR‐90, Wi‐38, and human fetal lung fibroblasts (here termed HFLF), primary lung fibroblasts (ATCC, CCL‐186, Coriell AG06814‐J, and Coriell AG06558, respectively), were cultured at 37°C in an atmosphere with 5% CO_2_ and 3% O_2_. All cells were cultured in DMEM complete media, which consisted of Dulbecco's Modified Eagle's Medium (DMEM) (Corning, 10‐013‐CV) supplemented with 10% (v/v) fetal bovine serum (FBS) (Millipore Sigma, F4135) and penicillin–streptomycin (Corning, 30‐001‐CI). To confirm the effect of UA on senescent cells, cells were treated 24 h after seeding (NS) or senescence induction (S‐dox) with 20 μM UA in complete growth media. The fresh media containing UA was replaced at least twice prior to analysis. All cells were mycoplasma‐free. Replicative senescence was induced by repeated passaging until PDL ≥ 59 or until cells stopped proliferating. NS cells were used until PDL 35. The cumulative PDL was calculated using the following equation:

$$\mathrm{PDL}=\frac{\log\;\left(\text{number of cells}\;\mathrm{at}\;\text{harvest}\right)-\log\left(\text{number of cells seeded}\right)\;}{\log\left(2\right)}$$

### 3.2 Primary Senescence Induction

Senescence was induced by treating cells with 300 nM doxorubicin hydrochloride (Millipore Sigma, 504042) for 24 h. After 24 h, doxorubicin was removed, and the cells were maintained in a complete medium for 9 days. On Day 9, the complete medium was changed to a low serum medium for 24 h before analysis.

### 3.3 Conditioned Medium (CM) Preparation and Secondary Senescence Induction

CM was generated by culturing primary senescent cells in low‐serum media for 24 h before harvesting and counting cells. Collected CM was spun down to remove cell debris, and the supernatant was stored at −80°C. All quantitative assays from CM were normalized to the cell number. Secondary senescence was induced by treating proliferating cells 24 h after seeding with 50% CM collected from primary senescent cells and 50% complete media for 5 days. The media was changed on Day 5, and CM and cells were collected, and multiple senescence markers were then tested.

### 3.4 Senescence‐Associated Beta‐Galactosidase

SA‐β Gal activity was measured using the Senescence Detection Kit (BioVison; K320), following the manufacturer's instructions. IMR90 cells were plated 1 day before senescence induction in 6‐well culture plates (Greiner Bio‐One; 657160). Non‐senescent cells were plated 3 days before staining. Staining was performed 10 days post‐senescence induction. During staining, cells were incubated for 48 h at 37°C without CO_2_ and then imaged by brightfield microscopy. The percentage of SA‐βGal‐positive cells was determined using ImageJ.

### 3.5 Viability Measurement

Cells were washed several times with PBS, fixed in 4% PFA, and stained with Hoechst (1:2000) for 10 min. Samples were then washed, and cells were imaged using a fluorescence microscope. The number of nuclei was counted using ImageJ software.

### 3.6 Immunofluorescence

IMR90 cells per well were plated in 96‐well plates, and senescence was induced in the plates as described above. NS cells were seeded 3 days before staining. All immunostaining was performed 10 days after treatment. Cells were fixed with 4% paraformaldehyde in PBS (Thermo Scientific, AAJ19943K2) for 15 min at room temperature. After washing with PBS, cells were permeabilized with 0.5% Triton X‐100 for 15 min, rinsed with PBS, and incubated overnight with primary antibody (1:1000 dilution with 5% BSA) at 4°C. Primary antibodies used included γH2AX mouse antibody (Novus Biologicals, NB100‐74435), pSTING rabbit antibody (Cell Signaling Technology, 19781S) and anti‐HMGB1 rabbit antibody (Abcam, ab18256). The following secondary antibodies were used: Alexa Fluor 488 goat anti‐mouse (Invitrogen, #A‐11008) and Alexa Fluor 546 goat anti‐rabbit (Invitrogen, #A‐11030). Cells were stained with Hoechst 33342 (1:2000; Invitrogen, H3570) in 5% BSA for 20 min at room temperature in the dark. Cells were washed with PBS before imaging. Cells with ≥ 2 γH2AX foci per nucleus and/or the absence of HMGB1 intracellularly were defined as senescent. Mean fluorescence intensity (MFI) was calculated per square area of a cell, subtracting background fluorescence, using ImageJ software.

Pico green (Thermo Fischer P11496) was combined with the mitochondrial dye—mito‐tracker red 633 for extranuclear DNA staining. Both dyes were added to cells in complete media for 1 h immediately before live cell imaging on a fluorescence microscope. Hoechst staining was used to separate nuclear staining from extranuclear foci. Pico green positive cells were manually counted, and their proportion in the total cell number was calculated as a percentage. At least five images were used for quantification per condition.

### 3.7 Mitoview Staining

Cells were stained with Mitoview Red 633 Dye (Biotium 70055) (50 nM), Mitoview Green Dye (Biotium 70054) (20 nM), and Hoechst (1:2000) for 30 min at 37°C in complete media. The media was replaced with PBS for live cell imaging immediately afterwards.

### 3.8 Quantitative Real‐Time PCR (Real Time‐qPCR)

RNA was extracted from cells using a Quick‐RNA Miniprep Kit (Zymo Research, R1055) according to the manufacturer's instructions. cDNA synthesis was performed using Takara PrimeScript RT Master Mix (Takara, RR036A) according to the manufacturer's instructions. Quantitative PCR was performed on a StepOnePlus Real‐Time PCR System using primers and probes purchased from Applied Biosystems TaqMan Gene Expression assays. Primers and probes used are listed in Table 1. All results are presented relative to the mean of housekeeping genes (ΔΔCt method), where target cDNA Ct values are normalized to those of actin.

**TABLE 1:** Thermo Fisher gene expression assay IDs for CDKN1A, CDKN2A, IL1A, IL6, IL8, CCL5, CCL2 used in the study.

	Thermo Fischer probe ID
CDKN1A	Hs00355782_m1
CDKN2A	Hs00923894_m1
IL1A	Hs00174092_m1
IL6	Hs00174131_m1
IL8	Hs00174103_m1
CCL5	Hs00174575_m1
CCL2	Hs00234140_m1

### 3.9 Enzyme‐Linked Immunosorbent Assays (ELISA)

Senescence was induced as indicated above, and the cells were cultured in low‐serum medium for 24 h before collection. CM was collected, and cell debris was removed by centrifugation at 300×g for 10 min. CM was analyzed with IL‐6 and IL‐8 ELISA kits (Millipore Sigma) as instructed by the manufacturer and normalized to the cell number.

### 3.10 Statistical Analysis

Data are presented as mean ± SEM. All statistical analyses were performed by one‐way ANOVA.

## Author Contributions

Amit Sharma, Manish Chamoli: conceptualization. Amit Sharma: supervision, funding acquisition. Anna Barkovskaya, Ashley Brauning, Aditi Thambala, Kriti Bhardwaj, Anand Rane: data collection. Anna Barkovskaya, Ashley Brauning, Aditi Thambala: formal analysis. Anna Barkovskaya, Ashley Brauning: writing – original draft preparation; Julie K. Andersen, Manish Chamoli, Amit Sharma: writing – review and editing.

## Conflicts of Interest

M.C. and J.K.A. are co‐founders of Symbiont Bio and declares no conflicts of interest.

## Supporting information


**Figure S1:** (A, B) Proportion of SA‐βGal‐positive Wi‐38 (A) and HFLF (B) NS and S‐dox cells with and without UA treatment. *n* = 3. (C) Relative viability of the NS and S‐dox cells normalized to respective untreated controls, with or without UA treatment in the indicated doses (5–40 μM). *n* = 3. (D) Number of γH2AX foci per nucleus in the NS and S‐dox IMR‐90 cells with and without UA treatment (*n* = 3). (E, F) IL6, IL8, and IL1α gene expression in HFLF (E), and Wi‐38 (F) NS and S‐dox cells with or without UA treatment. *n* = 3 (G) Representative image of the cells containing γH2AX extranuclear DNA foci. (H) Representative images of the NS and S cells with and without the UA treatment, labeled with DAPI, mito‐view tracker red, and pico green. Scale bar = 75 μm. (I) Representative images of the IMR‐90 NS cells and in cells treated with doxorubicin, evaluated at Days 1, 2, 5, and 7, labeled for pSTING and DAPI. Scale bar = 150 μm. **J**. pSTING fluorescence intensity quantification. *n* = 3. (K, L) Proportion of cells containing extranuclear pico green stained DNA foci in Wi‐38 (K), and HFLF (L) NS and S‐dox cells with or without UA treatment. *n* = 4. (M) Mitochondrial membrane potential assay. Red (potential‐dependent) to green (potential‐independent) staining intensity ratio in NS and S‐dox cells with or without the UA treatment. *n* = 3. All results are presented as a mean, and error bars represent SEM. Statistical analysis performed using one‐way ANOVA. *p < 0.033, **p < 0.002, ***p < 0.0002 ****p < 0.0001

## Data Availability

The data that support the findings of this study are available on request from the corresponding author.
