# Supplementary figures and images for: Mitigating Pro‐Inflammatory SASP and DAMP With Urolithin A: A Novel Senomorphic Strategy
Source: Aging Cell. 2025 Sep 29;24(11):e70237. doi: 10.1111/acel.70237 (PMC12608089; doi:10.1111/acel.70237)

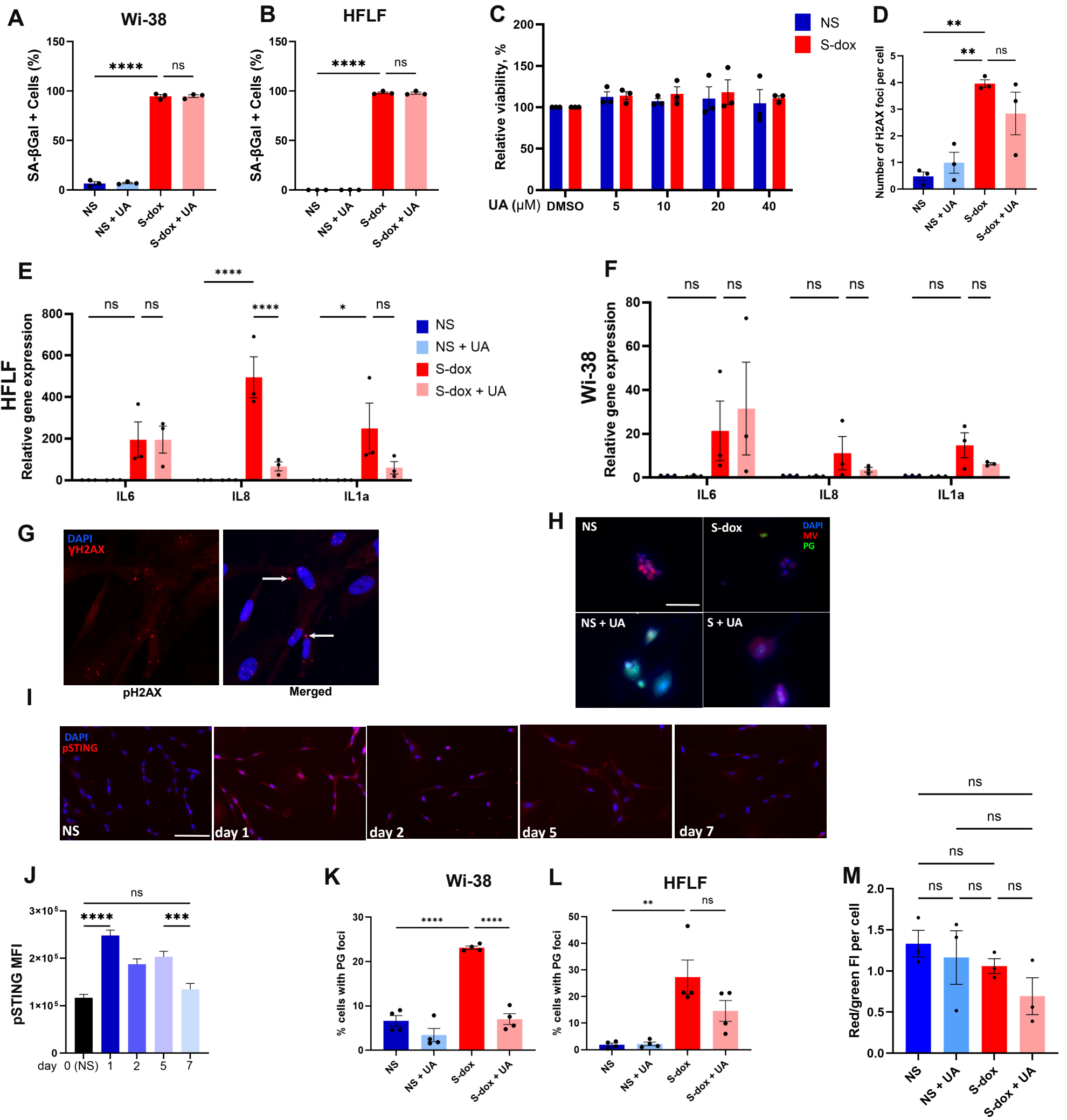

Supplement: Supplementary file 1 — Figure S1: (A, B) Proportion of SA‐βGal‐positive Wi‐38 (A) and HFLF (B) NS and S‐dox cells with and without UA treatment. n = 3. (C) Relative viability of the NS and S‐dox cells normalized to respective untreated controls, with or without UA treatment in the indicated doses (5–40 μM). n = 3. (D) Number of γH2AX foci per nucleus in the NS and S‐dox IMR‐90 cells with and without UA treatment (n = 3). (E, F) IL6, IL8, and IL1α gene expression in HFLF (E), and Wi‐38 (F) NS and S‐dox cells with or without UA treatment. n = 3 (G) Representative image of the cells containing γH2AX extranuclear DNA foci. (H) Representative images of the NS and S cells with and without the UA treatment, labeled with DAPI, mito‐view tracker red, and pico green. Scale bar = 75 μm. (I) Representative images of the IMR‐90 NS cells and in cells treated with doxorubicin, evaluated at Days 1, 2, 5, and 7, labeled for pSTING and DAPI. Scale bar = 150 μm. J. pSTING fluorescence intensity quantification. n = 3. (K, L) Proportion of cells containing extranuclear pico green stained DNA foci in Wi‐38 (K), and HFLF (L) NS and S‐dox cells with or without UA treatment. n = 4. (M) Mitochondrial membrane potential assay. Red (potential‐dependent) to green (potential‐independent) staining intensity ratio in NS and S‐dox cells with or without the UA treatment. n = 3. All results are presented as a mean, and error bars represent SEM. Statistical analysis performed using one‐way ANOVA. *p < 0.033, **p < 0.002, ***p < 0.0002 ****p < 0.0001 [file ACEL-24-e70237-s001.pdf]
